# Reverse epitope mapping of the E2 glycoprotein in antibody associated hepatitis C virus

**DOI:** 10.1371/journal.pone.0175349

**Published:** 2017-05-30

**Authors:** Amruta S. Naik, Ania Owsianka, Brendan A. Palmer, Ciaran J. O’Halloran, Nicole Walsh, Orla Crosbie, Elizabeth Kenny-Walsh, Arvind H. Patel, Liam J. Fanning

**Affiliations:** 1 Department of Medicine, University College Cork, Cork, Ireland; 2 MRC—University of Glasgow Centre for Virus Research, University of Glasgow, Glasgow, United Kingdom; 3 Department of Hepatology, Cork University Hospital, Cork, Ireland; 4 APC-Microbiome Institute, University College Cork, Cork, Ireland; Centre de recherche du CHUM, CANADA

## Abstract

The humoral immune system responds to chronic hepatitis C virus (HCV) infection by producing neutralising antibodies (nAb). In this study we generated three HCV pseudoparticles in which E1E2 glycoprotein sequence was targeted by the host humoral immune system. We used patient derived virus free Fabs (VF-Fabs) obtained from HCV genotype 1a (n = 3), genotype 1b (n = 7) and genotype 3a (n = 1) for neutralisation of HCVpp produced in this study both individually and in combination. Based on the available anti-HCV monoclonal nAb mapping information we selected amino acid region 384–619 for conformational epitope mapping. Amongst our notable findings, we observed significant reduction in HCVpp infectivity (p<0.05) when challenged with a combination of inter genotype and subtype VF-Fabs. We also identified five binding motifs targeted by patient derived VF-Fab upon peptide mapping, of which two shared the residues with previously reported epitopes. One epitope lies within an immunodominant HVR1 and two were novel. In summary, we used a reverse epitope mapping strategy to identify preferred epitopes by the host humoral immune system. Additionally, we have combined different VF-Fabs to further reduce the HCVpp infectivity. Our data indicates that combining the antigen specificity of antibodies may be a useful strategy to reduce (in-vitro) infectivity.

## Introduction

The humoral immune system reacts to Hepatitis C virus (HCV) infection by producing neutralising antibodies (nAb). However, it is often observed that in patients with persistent HCV infection, high titres of nAbs are developed but they fail to clear the infection. One of the reasons behind the failed humoral immune response is due to strain specificity of nAbs (reviewed in [1, 2]). Broadly neutralising monoclonal antibodies (bNAbs) which target conserved regions of the E1E2 glycoprotein have been shown to control HCV infection in cell culture and in animal models of HCV [3–5]. New data suggests that monoclonal antibodies (MAb) and polyclonal antibodies have the ability to provide protection against HCV infection (reviewed in [6, 7]). Virus like particles expressing E1E2 and E2 glycoprotein alone induced protective humoral immune response in chimpanzees [8]. Moreover, immunisation with recombinant E1E2 glycoprotein elicited cross-neutralising antibodies in chimpanzees, chimeric mice and healthy human volunteers [9–11].

Surface glycoproteins E1E2 are the major targets of nAbs as regions within these proteins facilitate interactions with host cell receptors during entry of HCV [12]. Glycoprotein E2 mediates protein-protein interactions with CD81 and scavenger receptor class B type I and is targeted by most of the nAbs and MAbs [12]. Several studies have shown that MAbs target amino acid residues 396–424, 436–447 and 523–540 in the E2 glycoprotein [2, 7, 13, 14]. Immunogenic E1E2 glycoprotein peptides, viral particles have been used to identify the epitopes targeted by bNAbs [15, 16]. The crystal structure of E2 glycoprotein has revealed that the N terminus of E2 glycoprotein harbours the broadly neutralising face (aa residues 412–453 and 503–535) [13, 17, 18]. It has been also observed that hypervariable region 1 (HVR1) located at the 5’ end of E2 is immunodominant, but nAb response to this region is strain specific and HVR1 acts as a decoy antigen [13, 19]. Various studies additionally have shown that mutation of the sites near nAb binding site can result into resistance to nAbs [5].

In our previous research we have demonstrated that viraemic sera can be segregated into antibody associated (AAV) and antibody free virus (AFV) fractions [20, 21]. We have observed that the host humoral immune system targets viral variants which are often clonotypic in nature [21–23]. Data from previous research has shown that total IgGs obtained from sera lacking detectable AAV were unable to target viral variants from the pool of quasispecies. These findings implied that the presence of AAV represents an active host immune response in the context of a complex serum based environment [24]. Based upon these findings we investigated the epitopes targeted by the host immune system in AAV.

In current study, we produced HCVpp from E1E2 sequences targeted by host humoral immune system. Our first goal was to investigate the infectivity of patient derived HCVpps and the neutralisation efficacy of VF-Fabs obtained from inter-genotype and inter-subtype patient sera. Secondly, we aimed to map the epitopes targeted by humoral immune system the E2 glycoprotein of AAV sequence. We focused our epitopes mapping to the E2 glycoprotein region 384–619 using VF-Fabs. Our epitope mapping data showed five different epitopes of which two have not been reported previously. We observed reduction in infectivity of pseudotyped particles expressing E1E2 isolated from AAV when challenged with a combination of unrelated VF-Fabs. Our study gives more insight into epitopes targeted by the host humoral immune system.

## Materials & methods

### Serum

This study was approved by Clinical Research Ethics Committee of the Cork Teaching Hospitals and written consent from patients was obtained. A panel of viraemic sera positive for HCV genotype 1a (n = 3), 1b (n = 7) and 3a (n = 1) were selected from chronically infected patients at different time points (Table 1). Six 1b serum samples were acquired from a cohort of Irish women infected with HCV genotype 1b via contaminated anti-D immunoglobulin [25]. The VERSANT® HCV Genotype Assay (LiPA, Siemens) was used to confirm the HCV genotype.

**Table 1:** Oligonucleotide Primers for site directed mutagenesis.

Primer Name	Primer Sequence
a655g I-V F	5'-GGAGACCCATACG**G**TAGGGGGGAGCGC-3'
a655g I-V R	5'-GCGCTCCCCCCTA**C**CGTATGGGTCTCC-3'
t677c V-A F	5'-CGCGAGCCGTGC**C**GCCCACCGCG-3'
t677c V-A R	5'-CGCGGTGGGC**G**GCACGGCTCGCG -3'
a367g T-A F	5'-tctctcagctgttc**G**ccttctcgcctcgc-3'
a367g T-A R	5'- gcgaggcgagaagg**C**gaacagctgagaga-3'

Bold letter indicates nucleotide site for mutation

### Fractionation of sera and glycoprotein cloning

The serum samples were fractionated into antibody associated virus (AAV) and antibody free virus fraction (AFV) using protein G sepharose columns (Ab Spin Trap™, GE Healthcare), as described previously [21]. RNA was extracted from first wash fraction (W0-AFV), the last wash fraction (W8) and elute containing Total IgG (AAV and free IgG) using QIAamp Viral RNA mini kit (Qiagen). cDNA sequences encoding full-length E1E2 were amplified from RNA extracted from Total IgG fraction using Expand High Fidelity PCR system (Roche) (Table 1) and cloned into the pcDNA3.1 V5his D-TOPO expression vector (Life Technologies) as previously described [26].

### HCVpp generation, infectivity and neutralisation assays

Cloned E1E2 genes were used to generate HCVpp, as described previously [27]. Plasmids used to generate HCVpp were obtained from INSERM, France. phCMV-ΔC/E1/E2 H77 (INSERM-TRANSFERT, France) was used as a positive control to generate HCVpp. Pseudoparticles generated in the absence of the E1E2 plasmid were used as a negative control. Infectivity assays were conducted using a luciferase readout as previously described [26, 27]. Infectivity assays were done in quadruplet with three technical replicate each. Statistical significance of HCVpp infectivity in comparison to no envelope control was determined via one-way analysis of variance (ANOVA) followed by Dunnett’s posthoc test. For neutralisation assays, pseudotyped virus were mixed with defined concentrations of virus free Fab (VF-Fab) ranging from 0.006–0.400 mg/ml (ND 1000, Spectrophotometer). VF-Fab were obtained from viraemic HCV sera which were classified as AAV positive upon initial fractionation. Briefly, the AAV positive sera were mixed with proteinase K (5 mg/ml) in 1:1 volume. The treated sera were then passed thorough Ab Spin Trap™ to collect the virus free Fab (VF-Fab) fraction [21]. Total IgG from proteinase K treated human serum from male AB plasma from three different lots were used as control (SLBK465 V, SLBF2588V, 051M0919, Sigma).VF-Fab-HCVpp were incubated for 1 h at 37°C, and then added to Huh7 cells [26]. Cultures were incubated for 72 h and the infectivity levels were determined by measuring luciferase activity in relative light units (RLU). Neutralisation assays were done in triplicate with duplicate technical replicates. The 50% inhibitory concentration (IC_50_) titre was calculated as the VF-Fab concentration that caused a 50% reduction in virus infectivity compared to the level in the virus control wells after subtraction of no envelope control RLU. All data were fitted using nonlinear regression variable slope with no constraint on hill slope (GraphPad Prism version 5).

### Site directed mutagenesis

We observed the two plasmids (1b-1-2 and 1b-1-3) coding for E1E2 sequence differed at amino acid positions 292 in the E1, 388 and 395 in the HVR1 domain. We generated six mutant strains of 1b-1-2 with a mutation for 1b-1-2_**I388V**,_ 1b-1-2_**V395A**_, _**T292A**_1b-1-2_**I388V**, **T292A**_1b-1-2_**V395A**,_ 1b-1-2_**DM**_ and _**T292A**_1b-1-2 _**DM**_ (DM corresponds to double mutant I_389_V and V_396_A) using primers enlisted in Table 1 (QuickChange Lightening Site Directed mutagenesis kit, Agilent Tech). HCVpp were then generated from two wild type 1b-1-2, 1b-1-3 and six mutants and infectivity assays were conducted following Bartosch *et al*. (2003) protocol [27].

Infectivity assays were done in quadruplet with three technical replicate each.

### Epitope mapping

A library of peptides beginning at the E2 N-terminus (residue 384 of the H77 reference strain AF011751) encompassing the first 236 amino acids of the protein was synthesized using chemically linked peptides on scaffolds (CLIPS) technology (Pepscan Presto; Lelystad, Netherlands) [28]. HCVpp1b-1-3 being highly neutralisation sensitive was chosen as a reference sequence for peptide synthesis. Four different peptide libraries were generated for the peptide microarray as following: (1) Linear peptides of 15 mer were derived from the target sequence with an offset of one residue, (2) Loop mimics of constrained peptides of length 17 were constructed (Pepscan Presto, Lelystad, Netherlands). Positions 2–16 were occupied by 15-mer sequences derived from the target sequence of HCV-E1E2 glycoprotein. To introduce structural constraints Cys were inserted on positions 1 and 17, which then were constrained by mP2 CLIPS, (3) Structured peptides of length 23 derived from the target sequence with an offset of one residue were synthesized to mimic the helical structure. Cys residues on positions 1 and 5 were joined by mP2 CLIPS and (4) Structured peptides of length 22 were constructed to mimic β-turn. 20-mer sequences on positions 2–21 were derived from the target sequence of HCV-E1E2 glycoprotein with an offset of one residue. “PG” (Pro, Gly) residues supplant the residues present on positions 10 and 11. Cys residues on positions 1 and 22 were joined by mP2 CLIPS. Native Cys were protected by acetamidomethyl in all the libraries.

The binding of VF-Fab isolated from 1a-1-3, 1b-1-3, 1b-5-1 and 3a-1-1 to each of the synthesized peptides was tested in a PEPSCAN-based ELISA [29]. The peptide arrays were incubated with primary antibody solution (overnight at 4°C). After washing, the peptide arrays were incubated with a 1/1000 dilution of goat anti-human HRP conjugate (Southern Biotech 2010–05) for one hour at 25°C. After washing, the peroxidase substrate 2,2’-azino-di-3-ethylbenzthiazoline sulfonate and 20 μl/ml of 3% H_2_O_2_ were added. After one hour, the colour development was measured. The colour development was quantified with a charge coupled device camera and an image processing system.

### SIFT analysis to study small genomic variations in putative epitopes in E2

We analysed the amino acid sequences of all the infectious HCVpp to assess whether an amino acid substitution in the predicted epitopes affect the protein function using a well characterised analytical tool called Sorting Intolerant From Tolerant (SIFT). SIFT calculates the probability based upon the position and physicochemical properties of an amino acid substitution having a deleterious effect on VF-Fab binding at any given position [30]. The threshold for functional intolerance to evaluate the amino acid substitution was 0.05.

## Results

### Diversity in HCVpp infectivity isolated from antibody associated virus

**Fig 1:**
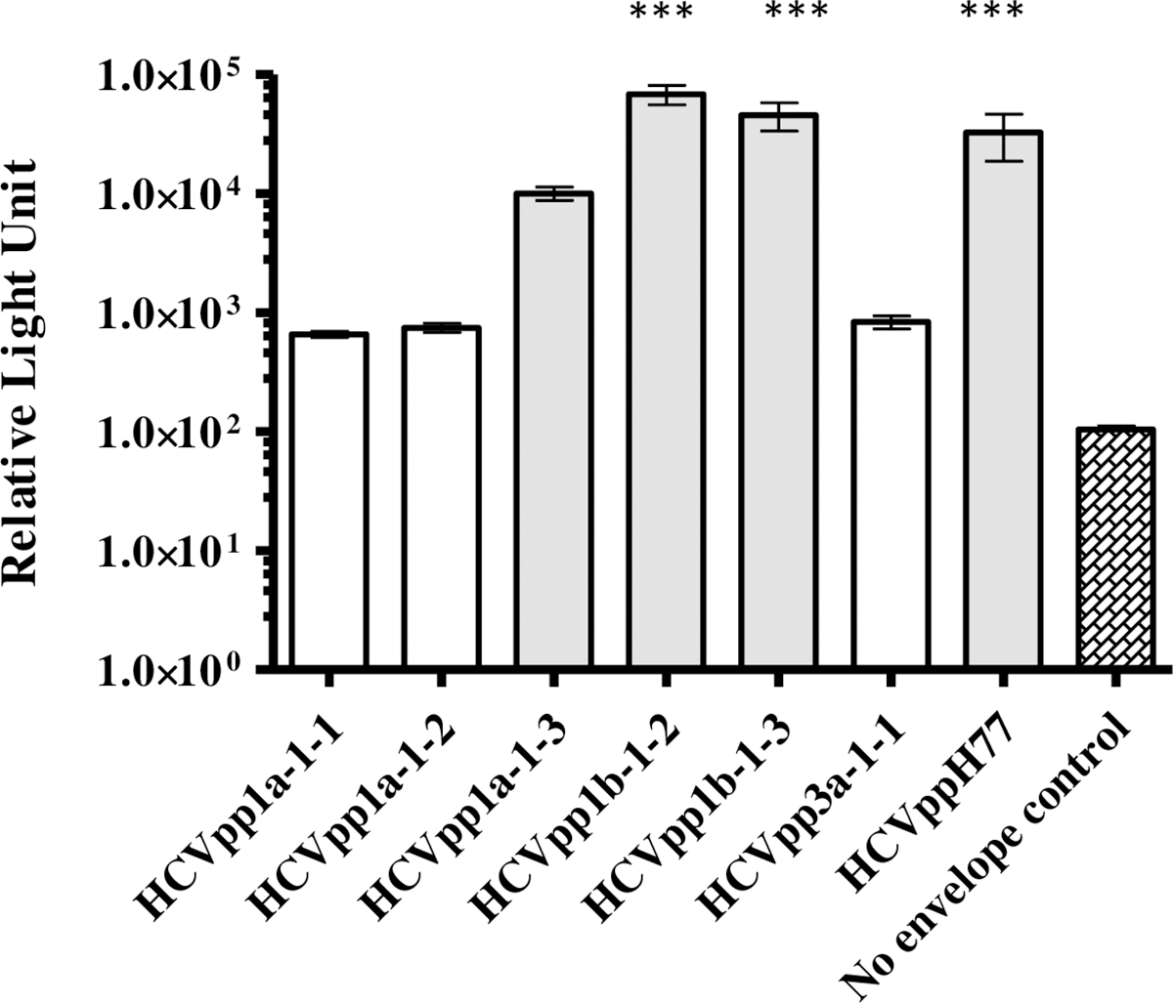
Varying degree of infectivity of E1E2 glycoproteins derived from AAV HCVpp. HCVpp generated from E1E2 associated with AAV showed varying degree of infectivity. HCVpp generated from phCMV-ΔC/E1/E2 H77 were used as reference HCVpp. No envelope control reproducibly gave RLU values less than 100, therefore a cut-off of 1000 RLU was used to determine the infectivity of a clone (***p<0.0001, one way ANOVA, Dunnett’s posthoc test) The X axis depicts infectious HCVpp clones (shaded boxes, n = 3/6) in this study. Y axis denotes infectivity in relative light units (RLU). GT: genotype

Serum samples obtained from five patients in this study were positive for AAV i.e., genotype 1a (n = 3), 1b (n = 4/7) and 3a (n = 1) (Table 2). We generated six E1E2 expression clones from these AAV. A threshold of 10 times greater relative light unit (RLU) than the no envelope control was set to identify infectivity of pseudoparticles [31]. We observed that E1E2 glycoproteins which were isolated from AAV when expressed in the HCVpp system showed a marked difference in their infectivity (Fig 1). HCVpp1a-1-1, HCVpp1a-1-2 and HCVpp3a-1-1 were non-infectious as they did not pass the threshold set for infectivity. According to recent work by Urbanowicz *et al*. (2016), the non-infectivity of these HCVpp could be due to the ratios of plasmids used in transfection and/or Murine Leukaemia virus packaging system [14]. However, the expressed E1E2 glycoproteins from the aforementioned HCVpp were recognised by MAb AP33 and patient derived virus free Fab (VF-Fab) in GNA capture ELISA (Data not shown). HCVpp1b-1-2 and HCVpp1b-1-3 generated highly infectious pseudoparticles (p<0.0001, ANOVA) while HCVpp1a-1-3 was infectious above the set threshold and hence was included in the neutralisation assay. (Fig 1).

**Table 2:** Sample characteristics used in the current study.

Genotype	No. of samples	AAV positive samples	Sample ^#^ identifier	Date of collection	Accession Number AAV	^$^VF-Fab
1a	3	+	1a-1-1	04/2007	KY031948	1a-1-1
		+	1a-1-2	08/2013	KY031950	1a-1-2
		+	1a-1-3	01/2014	KY031949	1a-1-3
1b	7	+	1b-1-2*	12/2013	KY031951	1b-1-2
		+	1b-1-3*	03/2014	KY031952	1b-1-3
		-	1b-4-1*	05/2014	N/A	1b-4-1
		+	1b-5-1	04/2015	KU888834^¥^	1b-5-1
		-	1b-6-1*	06/2014	N/A	1b-6-1
		-	1b-8-1*	10/2014	N/A	1b-8-1
		+	1b-10-1*	04/2015	KU888837^¥^	1b-10-1
3a	1	+	3a-1-1	12/2013	KY031953	3a-1

AAV: Antibody associated virus

^#^ Sample identifier: Genotype/Subtype-patient identifier- sample number

*Source of infection: contaminated anti-D immunoglobulin [25]

^¥^ AAV were obtained in previous study [24]

^$^ VF-Fab—Virus free Fab obtained from sera positive for AAV complex

### Mutations at 292 in the E1 and 388 in the HVR1 of glycoprotein related to infectivity of HCVpp1b-1-3

**Fig 2:**
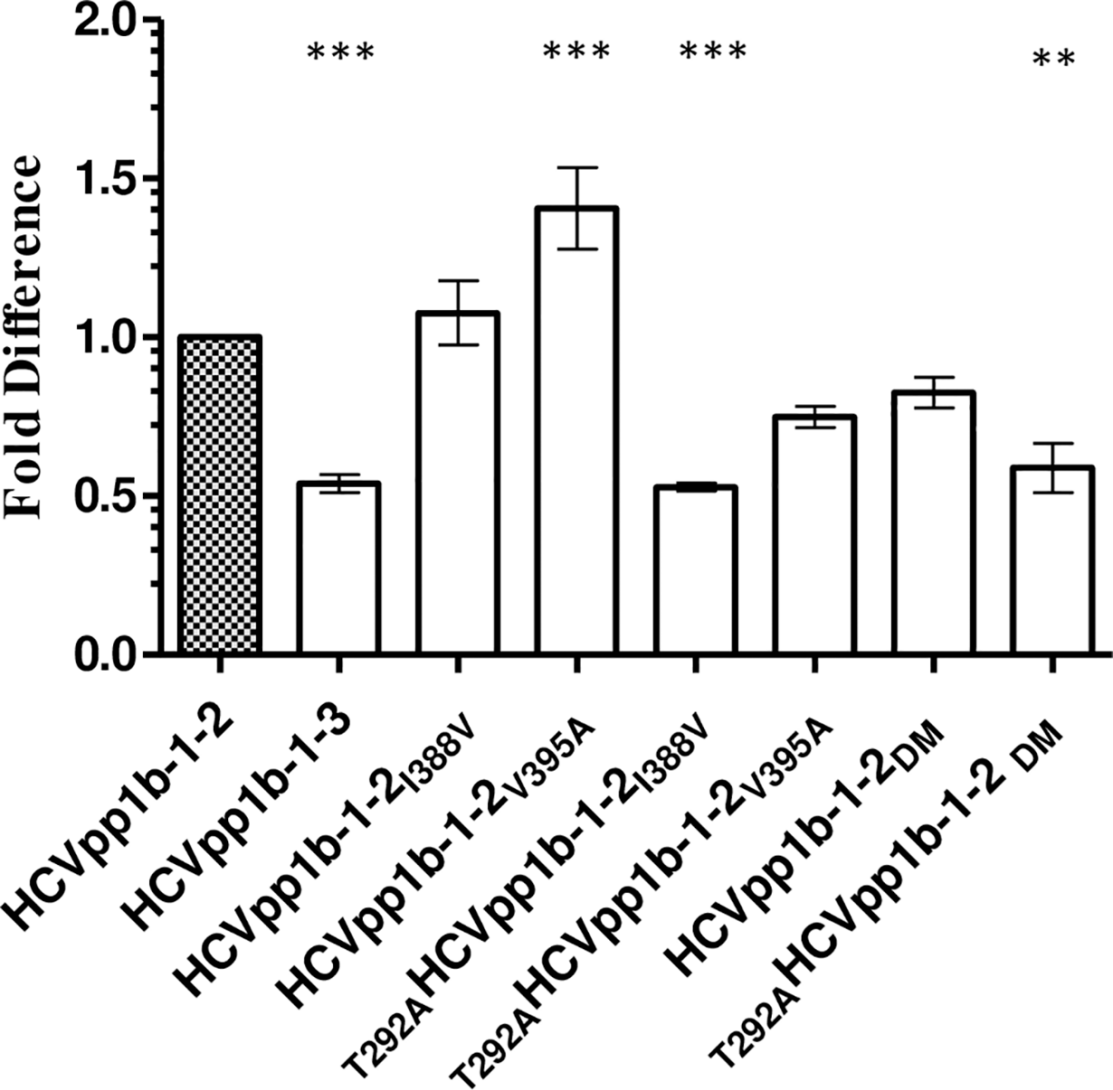
Mutation in the E1 glycoprotein affects the infectivity of HCVpp. Six mutant clones were generated for highly infectious HCVpp1b-1-2 as follows, 1b-1-2_I388V,_ 1b-1-2_**V395A**_, _**T292A**_1b-1-2_I388V, T292A_1b-1-2_V395A,_ 1b-1-2_**DM**_ and _**T292A**_1b-1-2_**DM**_ (DM corresponds to double mutant I_**388**_V and V_**395**_A). Wild type clones HCVpp1b-1-2 and HCVpp1b-1-3 were used as controls. As a reference infectivity value of HCVpp1b-1-2 was set to 1. Mutation in the E1 glycoprotein reduces the infectivity of HCVpp1b-1-2. Pairwise mutation at position 292 and 388 (_**T292A**_1b-1-2_**I388V**_) and _**T292A**_1b-1-2_**DM**_ corresponds to wild type HCVpp1b-1-3 (*p<0.0001, one way ANOVA, Dunnett’s posthoc test). The X axis depicts infectious mutant HCVpp clones in this study. Y axis denotes % infectivity.

We observed that HCVpp1b-1-2 was average 2 fold more infectious than HCVpp1b-1-3 (Fig 2, p<0.0001). It is interesting to note that these 1b HCVpp expressing E1E2 glycoproteins were obtained from the same patient at two different time points (Table 2). HCVpp1b-1-2 differed from HCVpp1b-1-3 by only three amino acids at positions 292 (C-terminus of E1), 388 and 395 (HVR1 of the N-terminal of E2) in the E1E2 glycoprotein sequence and yet showed differences in the infectivity (Fig 1). We hypothesized that these three amino acid co-ordinates were responsible for the observed differences in the infectivity. As a reference range, infectivity of HCVpp1b-1-2 was set to 1. Statistical significance of HCVpp infectivity in comparison to HCVpp1b-1-2 was determined via one-way analysis of variance (ANOVA) followed by Dunnett’s posthoc test. Individual mutants, 1b-1-2_**I388V**_ and 1b-1-2_**V395A**_, generated 0.11 fold and 0.40 fold (p<0.0001) greater levels of infectious particles compared to that of HCVpp1b-1-2 respectively (Fig 2). These results indicated that alanine substitution at position 395 plays a more significant role in enhancing HCVpp infectivity *in vitro* than wild type valine. A mutation in the E1 at T292A dramatically decreased the HCVpp infectivity by 0.6 fold for _**T292A**_HCVpp1b-1-2_**I388V**_ and 0.7 fold (p<0.0001) for _**T292A**_HCVpp1b-1-2_**V395A**_ in comparison to the individual mutant clones HCVpp1b-1-2_**I388V**_ and HCVpp1b-1-2_**V395A**_. These results indicate a potential role for the amino acid at position 292 (within E1) in governing the degree of infectivity *in vitro* (Fig 2). Importantly, the _**T292A**_1b-1-2_**DM**_ clone (p<0.001) closely replicates the infectivity of the HCVpp1b-1-3 wild type.

### Neutralisation of antibody associated E1E2 HCVpp

**Fig 3:**
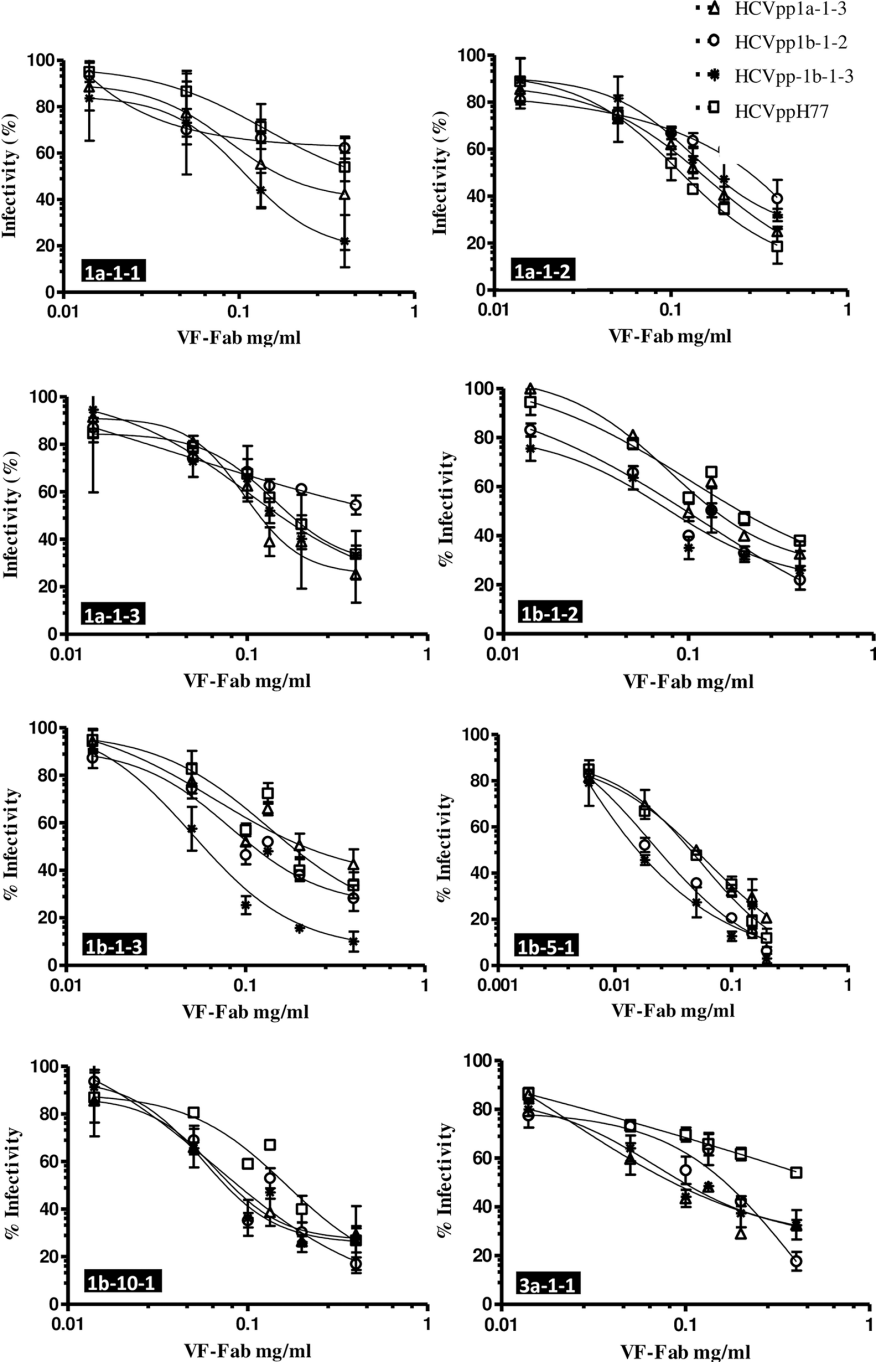
Patient derived VF-Fab display varying degree of neutralising ability to different E1E2 HCV pseudoparticles. VF-Fab were purified from antibody-virus complex. VF-Fab1a-1-1, VF-Fab1a-1-2, VF-Fab 1a-1-3 were purified from a patient infected with HCV genotype 1a. VF-Fab1b-1-2, VF-Fab1b-1-3 and VF-Fab1b-10-1 were purified from a patients infected with genotype 1b whose common source of infection was HCV infected anti D immunoglobulin [25]. VF-Fab1b-5-1 were purified from a blood transfusion patient infected with genotype 1b. VF-Fab3a-1-1 was purified from a patient infected with HCV genotype 3a (Table 1). HCVpp incorporating E1E2 derived from genotype 1a (HCVpp1a-1-3, HCVppH77), 1b (HCVpp1b-1-2 and HCVpp1b-1-3) were pre-incubated with different concentrations (0.006 to 0.4 mg/ml) of purified VF-Fabs prior to infection of Huh7 cells. No envelope control was used to normalise the data. The neutralising activity of the VF-Fab is expressed as percentage of inhibition of the infectious titres. VF-Fab1b-5-1 bottom values were constraint to zero for that data set only. Each experiment was repeated three times. IC_50_ for each VF-Fab is detailed in Table 3. Error bars indicate standard deviation.

In our previous research we have shown that patient derived VF-Fab target clonotypic viral variant in the quasispecies population [24]. In order to determine the neutralisation potency of patient derived VF-Fab, AAV positive source sera and control sera were treated with proteinase K as described earlier [21, 24]. Eight VF-Fabs, from genotype 1a (n = 3), genotype 1b (n = 4), genotype 3a (n = 1) and control (n = 3) were each tested for neutralisation of HCVppH77, HCVpp1a-1-3, HCVpp1b-1-2 and HCVpp1b-1-3. The IC_50_ are presented in Table 3 and the corresponding neutralisation curves are presented in Fig 3. We observed that the panel of patient derived VF-Fab cross-neutralise genotype 1 HCVpp efficiently. Post proteinase K treated Total IgG from control sera did not neutralise the HCVpp in our panel (S1 Fig). VF-Fab1a-1-2, VF-Fab1b-1-2, VF-Fab1b-1-3, VF-Fab1b-5-1 and VF-Fab1b-10-1 showed greatest neutralisation breadth, reducing by at least 50% of the infection for all the HCVpp clones (Fig 3). The sensitivity to each VF-Fab varied across the HCVpp panel, with HCVpp1b-1-3 being highly sensitive (relative infection range 2–32%), HCVpp1a-1-3, HCVpp1b-1-2 being moderately resistant (relative infection range 10–65%) to each of the tested VF-Fab. HCVpp generated from phCMV-ΔC/E1/E2 H77 were resistant to the VF-Fab1a-1-1 and VF-Fab3a-1-1 neutralisation and showed varying degree of sensitivity to the VF-Fab1a-1-2, VF-Fab1a-1-3, VF-Fab1b-1-2, VF-Fab1b-1-3 and VF-Fab1b-10-1 (Fig 3). VF-Fab1b-5-1 at the concentration of 0.200 mg/ml and VF-Fab1b-10-1 at 0.400 mg/ml were highly neutralising inhibiting the infectivity of all the HCVpp clones by 75–96% (Fig 3). Despite showing highest neutralisation potential, unique fit for VF-Fb1b-5-1 was not identified. This can be corrected by including either higher concentration of VF-Fab or constraining the values for the top and/or bottom parameters (GraphPad Prism guidelines). However, at 200 μg/ml concentration VF-Fab1b-5-1 reduced the infectivity by average 80–98% in HCVpp. In this situation using concentration higher than 200 μg/ml was not ideal. Hence, values for the bottom parameter for VF-Fab1b-5-1 were constrained to zero.

**Table 3:** IC_50_ values (mg/ml) of neutralisation curves in Fig 3 and Fig 4 for each VF-Fab.

HCVpp	1a-1-1	1a-1-2	1a-1-3	1b-1-2	1b-1-3	1b-4-1	1b-5-1*	1b-6-1	1b-8-1	1b-10-1	3a-1-1
1a-1-3	0.088	0.161	0.097	0.080	0.067	-	0.061	0.038	-	0.066	0.023
1b-1-2	-	-	0.041	0.117	0.081	0.087	0.021	0.087	-	0.066	0.399
1b-1-3	0.108	0.133	0.100	0.075	0.036	-	0.015	-	0.132	0.057	0.069
H77	0.142	0.111	0.140	0.100	0.134	-	0.054	-	0.235	0.165	-

*IC_50_ values were obtained by constraining bottom to zero.

### Total IgG derived from sera without detectable AAV shows neutralisation activity

**Fig 4:**
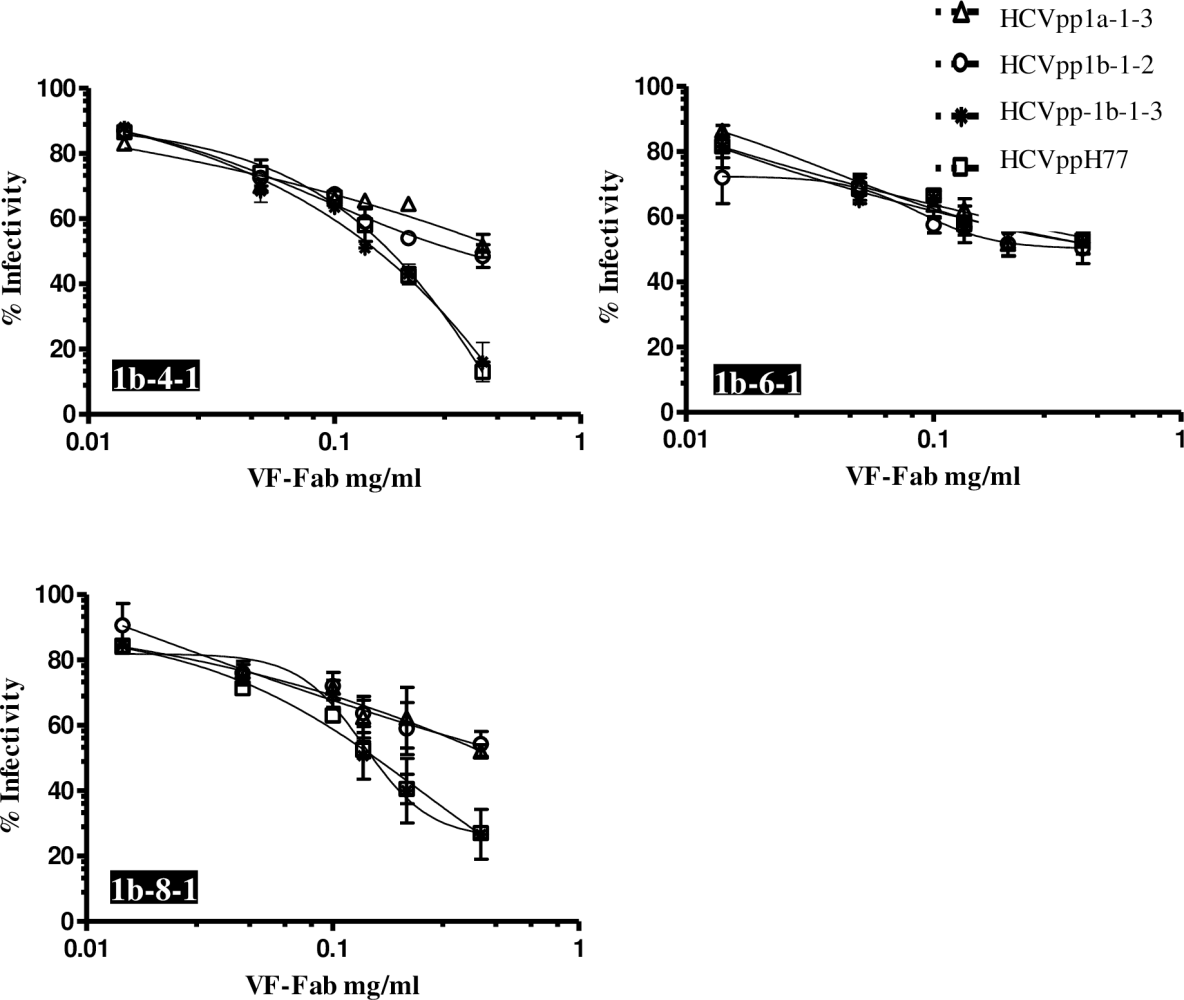
VF-Fab from sera without detectable AAV shows neutralisation activity. VF-Fab1b-4-1, VF-Fab1b-6-1, VF-Fab1b-8-1 were purified from three unrelated patients infected with HCV genotype 1b that were negative for presence of AAV. HCVpp incorporating E1E2 derived from genotype 1a (HCVpp1b-1-3, HCVppH77) and 1b (HCVpp1b-1-2, HCVpp1b-1-3) were pre-incubated with different concentrations (0.006 to 0.4 mg/ml) of purified VF-Fabs prior to infection of Huh7 cells. A no envelope control was used to normalise the data. The neutralising activity of the VF-Fab is expressed as percentage of inhibition of the infectious titres. Each experiment was repeated three times. IC_50_ for each VF-Fab is detailed in Table 3. Error bars indicate standard deviation.

In our previous research, we observed that Total IgG purified from sera classified as AAV negative do not show viral variant targeting activity in the viraemic HCV sera [24]. In the current work, we tested the ability of Total IgG from AAV negative sera to neutralise HCVpp. We treated the AAV negative sera with proteinase K and obtained Fabs as described previously [24]. We observed that HCVpp1b-1-3 and HCVppH77 were the highly neutralisation sensitive to VF-Fab1b-4-1 and VF-Fab1b-8-1, reducing the infectivity by 75% and 85% respectively (Fig 4). However, HCVpp1a-1-3 and HCVpp1b-1-2 were resistant to neutralisation by VF-Fab1b-4-1, VF-Fab1b-6-1 and VF-Fab1b-8-1 (Fig 4). Although the VF-Fab were derived from sera from an anti-D cohort, we observed varying degree of neutralisation breadth in HCVpp1b-1-2 and 1b-1-3, which were also derived from an anti-D patient serum.

### Conformational epitope mapping of E2

**Fig 5:**
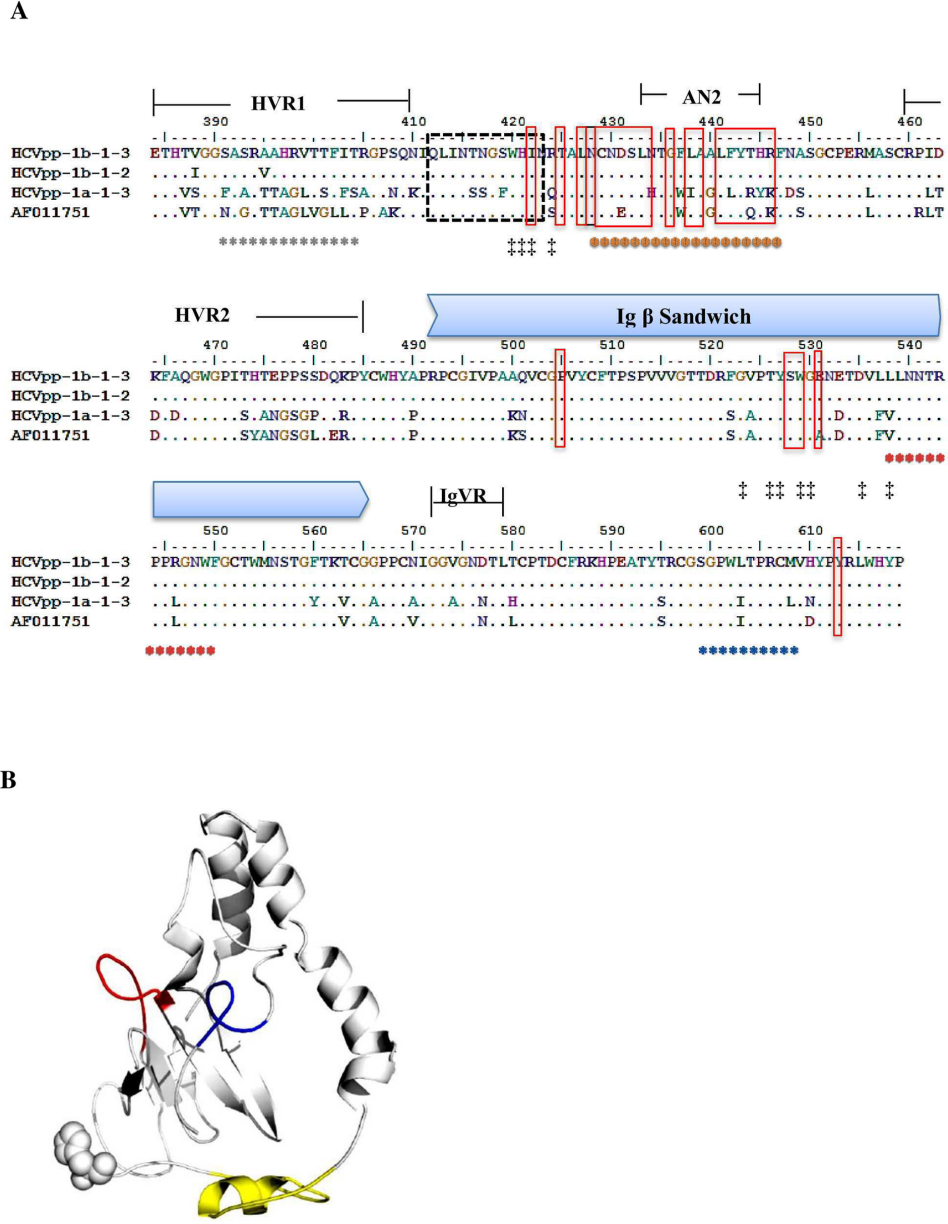
Conformational epitope mapping of E2 glycoprotein region starting from amino acid 384–619. **A.** The multiple sequence alignment (MSA) of the E2 glycoprotein. HCVpp1b-1-3 is a reference sequence; HCVpp1b-1-1, HCVpp1a-1-3 and HCVppH77 were the infectious pseudoparticles. AF011751 is an H77 strain. aa marked with *: targeted by patient derived VF-Fabs. The colour code of the epitopes represent the position of the epitope in 3D structure of E2 glycoprotein (Table 4); aa in dotted box: linear epitope targeted by AP33 mouse MAb [35]; aa in red box: residues on E2 interact with AR3C HuMAb [13]; aa marked with ‡: residues important for CD81 binding [13]. **B.** The 3D model of HCV-E1E2 glycoprotein shown in white cartoon with flexible non-modelled E2 N-termini (384–412) labelled with spheres. Sequence _428_NCNDSLNTGFLAALFYTHRF_447_ is highlighted in yellow, _539_LLNNTRPPRGNWF_550_ in red and _599_SGPWLTPRCM_608_ in blue (Pepscan Presto; Lelystad, Netherlands)

Based on the available anti-HCV monoclonal nAb mapping information and E2 structure, we selected amino acid residues that cover HVR1 (384–410), HVR2 (460–482), E2 β-sandwich (492–566) and IgVR (572–588) spanning up to aa 619 for conformational epitope mapping [2, 13, 17, 19, 32, 33]. Five different binding motifs were targeted by VF-Fab1a-1-3, VF-Fab1b-1-3, VF-Fab1b-5-1 and VF-Fab3a-1-1 in HCVpp1b-1-3 sequence upon peptide mapping (Table 4, S2 Fig). Amino acid variation in the epitopes targeted by patient derived VF-Fabs in infectious HCVpps from this study are shown in Fig 5A. The 3D model of sequence mapped for potential epitopes (HCVpp1b-1-3) is shown in Fig 5B. Our epitope mapping data showed that all the VF-Fab targeted an immunodominant epitope AN1 within HVR1 (Table 4, S2 Fig). The regions within or located near HVRs are assumed to be flexible loops (VR1, VR2 and IgVR) [34]. Of note, VF-Fab1b-5-1, VF-Fab3a-1-1 showed a higher binding affinity towards the β-turn mimics of AN1_393-405_ as compared to the linear mimics by VF-Fab1a-1-3 and VF-Fab1b-1-3. We also found that epitope AN2_433-445_ which overlaps with AN3_428-447_ is a part of discontinuous B cell epitope targeted by MAb AR3C (428–433, 436, 438, 439, 441–443, 446) [13, 17, 18]. Studies involving mutagenesis of HCV E2 have proposed three CD81 binding regions [34]. Notably, epitope AN5_599-608_ which lies beside IgVR_572-588_ [19] has not been reported previously.

**Table 4:** Putative Epitopes targeted by VF-Fab.

VF-Fab	Motif	Epitope Name
1a-1-3,1b-1-3,1b-5-1^*^, 3a-1-1^*^	_393_SRAAHRVTTFITR_405_	AN1^#^
1a-1-3	_433_LNTGFLAALFYTH_445_	AN2
1b-5-1^*^, 3a-1-1^*^	_428_NCNDSLNTGFLAALFYTHRF_447_	AN3
1b-1-3, 1b-5-1^*^, 3a-1-1^*^	_539_LLNNTRPPRGNWF_551_	AN4
3a-1-1	_599_SGPWLTPRCM_608_	AN5

*VF-Fabs binding to β-turn mimics was higher when compared with that of linear mimics [Ref AF011751] (Pepscan Presto; Lelystad, Netherlands)

^#^ AN1 epitope is located within HVR1 which is non-modelled and is represented in a white spherical cartoon (Fig 5B).

### Combination of VF-Fab significantly reduces HCVpp infection

**Fig 6:**
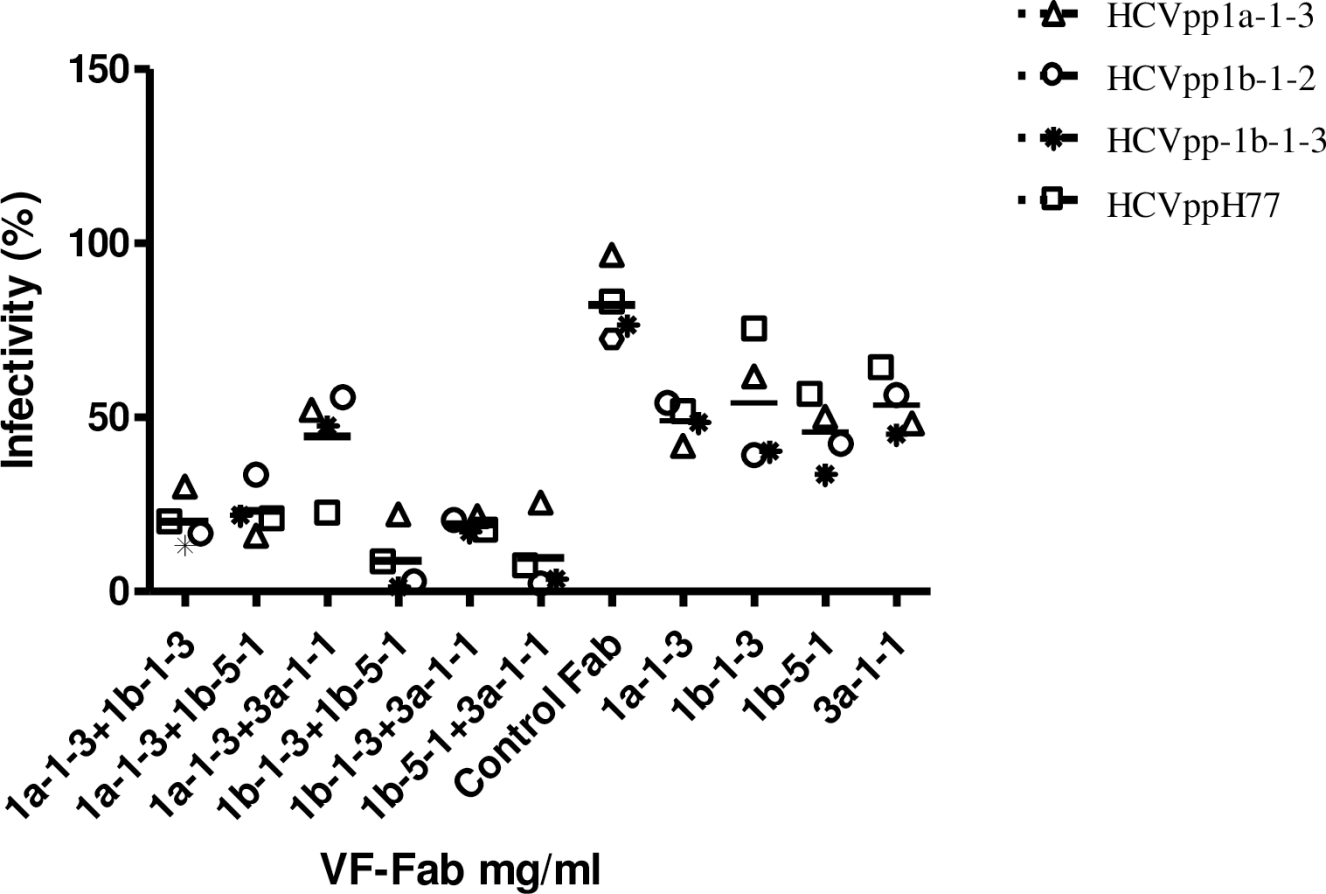
Neutralisation of pseudotyped virus with combination of VF-Fabs. HCVpp1a-1-3, HCVpp1b-1-2, HCVpp1b-1-3 and control HCVppH77 were tested for neutralisation using the average Log IC_50_ (mg/ml) for the VF-Fab in combinations (Table 5). The data is obtained from three independent experiments. Data for all the HCVpp were grouped together for statistical analysis. Significance was tested using one-way ANOVA with Dunnett t test (p<0.05–0.001, Table 5). Each VF-Fab combination was compared against individual VF-Fab at the concentration used in combination experiments.

Based on the date of collection of sera, IC_50_, and epitope mapping data (Tables 1 and 3), we selected VF-Fab1a-1-3, VF-Fab1b-1-3, VF-Fab1b-5-1 and VF-Fab3a-1-1 for our mixed VF-Fab challenge. The ratio of the combined VF-Fabs was set by choosing the average LogIC_50_ for each of the selected VF-Fab (Prism 5 guide lines). We combined RLU for all the HCVpp treated with a particular VF-Fab combination for statistical analysis. We observed a significant reduction in HCVpp infectivity with VF-Fab1a-1-3 and VF-Fab1b-1-3, VF-Fab1a-1-3 and VF-Fab1b-5-1, VF-Fab1b-1-3 and VF-Fb1b-5-1 and VF-Fab1b-5-1 and VF-Fab3a-1-1 combinations when compared to the individual VF-Fab concentration (Dunnett t test, p<0.05–0.001) (Table 5, Fig 6). However, VF-Fab1a-1-3+3a-1-1 in combination did not show any substantial reduction (in infectivity) compared to individual VF-Fab concentration (Table 5, Fig 6).

**Table 5:** Summary of statistical significance of average IC_50_ (mg/ml) of VF-Fab in combination in comparison with the individual VF-Fab.

VF-Fabs	1a-1-3	1b-1-3	1b-5-1	3a-1-1
Average concentration mg/ml	0.078	0.078	0.02	0.108
1a-1-3+1b-1-3	**	***	**	***
1a-1-3+1b-5-1	**	**	*	**
1a-1-3+3a-1-1	ns	ns	ns	ns
1b-1-3+1b-5-1	***	***	***	***
1b-1-3+3a-1-1	**	***	**	***
1b-5-1+3a-1-1	***	***	***	***
1a-1-3	n/a	ns	ns	ns
1b-1-3	ns	n/a	ns	ns
1b-5-1	ns	ns	n/a	ns
3a-1-1	ns	ns	ns	n/a

*p<0.05

**p<0.001

***p<0.0001, ns = not significant, n/a = not applicable

### Analysis of tolerated amino acid substitution in predicted motifs

Using an *in silico* SIFT analysis, we discovered that epitopes AN2-AN5 were not affected by the (observed) variation at the different positions in the epitopes (Table 4). Amino acid residues in the binding motifs of AN2-AN5 were broadly conserved. It is likely that amino acid variations in aforesaid epitopes of HCVpp sequences were functionally tolerable and hence, were recognised by the VF-Fab in this study (deleterious variations <0.05, Tables 6–9). However, in case of AN1, only an amino acid substitution at position 395 was predicted as tolerable with respect to the VF-Fab binding (Tables 6–9). AN1 lies in the HVR1, an immunodominant region which generates strain specific immune response.

**Table 6:** Tolerance to the amino acid substitution in the epitopes targeted by VF-Fab1a-1-3.

AN1												
_393_S	R	A	A	H	R	V	T	T	F	I	T	_405_R
A	-	T	T	A	G	L	V	G	L	F	S	A
G	-	V						S		L		P
-	-	1	-	-	-	-	-	-	-	-	-	-
1	-	0.34	1	1	1	1	0	1	1	1	1	1
0	-	1	0	0	0	0	0	0	0	0	0	0

Data presented is analysed and predicted using programme SIFT (http://sift.jcvi.org/www/SIFT_aligned_seqs_submit.html). Row corresponds to a position in the reference epitope. Column corresponds to amino acid variation (observed in our data set) at that position in different HCVpp (Fig 4A). Score at a particular position for an amino acid substitution is mentioned below the amino acid variant. The default threshold for functional intolerance used was 0.05 for amino acid substitution.

**Table 7:** Tolerance to the amino acid substitution in the epitopes targeted by VF-Fab1b-1-3.

AN3																			
_428_N	C	N	D	S	L	N	T	G	F	L	A	A	L	F	Y	T	H	R	_447_F
			E			H			W	I		G		L		Q	Y	K	
																R			
-	-	-	1	-	-	1	-	-	1	1	-	1	-	1	-	1	1	1	-
-	-	-	0.73	-	-	0.46	-	-	0.94	0.61	-	1	-	1	-	0.65	0.68	1	-
-	-	-	-	-	-	-	-	-	-	-	-	-	-	-	-	0.8	-	-	-

Data presented is analysed and predicted using programme SIFT (http://sift.jcvi.org/www/SIFT_aligned_seqs_submit.html). Row corresponds to a position in the reference epitope. Column corresponds to amino acid variation (observed in our data set) at that position in different HCVpp (Fig 4A). Score at a particular position for an amino acid substitution is mentioned below the amino acid variant. The default threshold for functional intolerance used was 0.05 for amino acid substitution.

**Table 8:** Tolerance to the amino acid substitution in the epitopes targeted by VF-Fab1b-5-1.

AN4												
_539_L	L	N	N	T	R	P	P	R	G	N	W	_551_F
V								L				
1	-	-	-	-	-	-	-	1	-	-	-	-
1	-	-	-	-	-	-	-	1	-	-	-	-

Data presented is analysed and predicted using programme SIFT (http://sift.jcvi.org/www/SIFT_aligned_seqs_submit.html). Row corresponds to a position in the reference epitope. Column corresponds to amino acid variation (observed in our data set) at that position in different HCVpp (Fig 4A). Score at a particular position for an amino acid substitution is mentioned below the amino acid variant. The default threshold for functional intolerance used was 0.05 for amino acid substitution.

**Table 9:** Tolerance to the amino acid substitution in the epitopes targeted by VF-Fab3a-1-1.

AN5									
_599_S	G	P	W	L	T	P	R	C	_608_M
				I					L
-	-	-	-	1	-	-	-	-	1
-	-	-	-	1	-	-	-	-	1

Data presented is analysed and predicted using programme SIFT (http://sift.jcvi.org/www/SIFT_aligned_seqs_submit.html). Row corresponds to a position in the reference epitope. Column corresponds to amino acid variation (observed in our data set) at that position in different HCVpp (Fig 4A). Score at a particular position for an amino acid substitution is mentioned below the amino acid variant. The default threshold for functional intolerance used was 0.05 for amino acid substitution.

## Discussion

In current study, we identified five epitopes which were targeted by human humoral immune system. We are the first to use E1E2 sequence isolated from AAV for epitope mapping, using patient derived VF-Fab. Furthermore, we have shown that targeting multiple E2 epitopes using patient derived VF-Fabs significantly reduces HCVpp infectivity.

In our previous research, we have shown that the host immune system targets discrete viral variants within the quasispecies [20, 21, 23]. In this study, we generated HCVpp from AAV E1E2 sequences. A recent study by Urbanowicz *et al*. (2015) noticed marked difference in the infectivity pattern of closely related clones in HCVpp system [31]. In their recent work, Urbanowicz *et al*. (2016) observed a number of factors to be responsible for the infectivity of HCVpp as followed i) differences in the amounts of plasmid used ii) species of packaging construct (an appropriate retrovirus backbone) iii) balancing the delivery of plasmids encoding the packaging vector and glycoproteins [14]. We also acknowledge that the E1E2 expressed by expression vector and proper folding of the glycoprotein could also influence the infectivity of the HCVpp. We made similar observations in terms of infectivity of HCVpp (Fig 1). However, we observed that HCVpp1b-1-2 and HCVpp1b-1-3 were closely related yet differed in their infectivity. Recently, Urbanowicz *et al*. (2016) have shown that difference in the infectivity of closely related E1E2 clones is due to isolate specific mutations in these genes [14]. Our site directed mutagenesis study showed that mutations in the HVR1 of E2 were restricted and maintained the physicochemical properties by replacing hydrophobic amino acid (V395A and I388V) and thereby infectivity of HCVpp (Fig 2). Pe´rez-Berna *et al*. (2006) showed that residues 265–296 in the E1 glycoprotein are hydrophobic and have membranotropic characteristics making them a candidate for membrane fusion [36]. We observed that in the E1 glycoprotein of HCVpp1b-1-3, a polar amino acid (T) was replaced by a hydrophobic (A) amino acid at position 292 (SIFT score 0.26). Supplementary screening of HCV genotypes from GenBank revealed that position 292 is conserved (First 123 hits, Data not shown). We also observed that pairwise mutation at position E1_292_ and E2_388_ lead to decrease in the HCVpp infectivity similar to the clone harbouring all three mutations. Western blot analysis of HEK extracts (from which HCVpp were generated) showed decreased expression of E2 glycoprotein in HCVpp1b1-1-2 mutants harbouring T292A substitution (Data not shown). However, it has been observed that expression of glycoprotein cannot be directly correlated with the infectivity [14]. In the absence of well-defined structure for E1 and the hypervaraible region in the E2, our data provides an insight into probable engagement of E1_292_ and E2_388_ in the infectivity of viral particle at least in HCVpp1b-1-3 (Fig 2). In this instance, our data supports the hypothesis that these amino acid coordinates play an important role in the infectivity of HCVpp1b-1-3.

We investigated neutralisation efficacy of VF-Fabs against the HCVpp expressing patient derived glycoprotein. In the case of genotype 1a, we observed that VF-Fab1a-1-1, VF-Fab1a-1-2 and VF-Fab1a-1-3 were able to neutralise HCVpp1a-1-3, all were acquired from the same patient at different time points (Table 2, Fig 3). We made similar observations for VF-Fab1b-1-2 and VF-Fab1b-1-3, which had ability to neutralise HCVpp1b-1-2 and HCVpp1b-1-3 (Fig 3). Neutralisation of HCVpp by the VF-Fab derived from the earlier serum samples (Table 2) suggests that the viral escape from the humoral immune response continues even after years of chronic infection. Most significantly, we also detected that VF-Fabs from unrelated patient sera 1b-5-1, 1b-10-1 and 3a-1-1 were efficient in reducing the HCVpp infectivity in all cases, suggesting that these VF-Fabs were broadly neutralising.

Of note, when we used proteinase K treated Total IgG from these AAV negative sera, we observed neutralisation activity in HCVpp system. These latter sera likely have nAbs against now extinct viral variants, which, targeted conserved epitopes present in the homogenous HCVpp system of HCVpp1b-1-3 and HCVppH77 (Fig 4).

A recent study on H5N1 strain of influenza virus has shown promising use of neutralising antibodies as prophylactic and therapeutic agents [37]. A cocktail of ZMab and MB003 (ZMapp—c13C6, c2G4, and c4G7) has been shown to provide 100% protection in nonhuman primates post 5 days of EBOV infection [38]. Bukh *et al*. (2015) have shown that polyclonal antibodies purified from chronic HCV patient can supress the homologous virus in chimpanzee for 18 weeks [39]. However, it failed to provide protection against the heterologous strains. These studies collectively show the value of using an antibody adjunct to achieve control of viraemia.

In our study, we observed that VF-Fabs when used in a combination had an enhanced effect in reduction of HCVpp infection (Fig 5). We posited the following question, is this decline in the infectivity of HCVpp due to multiple E2 epitopes targeting? We investigated this using CLIPS technology for epitope mapping. Here we used E1E2 sequence from HCVpp1b-1-3 targeted by host immune system which was highly neutralisation sensitive (Fig 3). Our study demonstrated that all the VF-Fabs used in the combination experiment recognised epitope AN1_393-405_ within HVR1 of E2 glycoprotein (Fig 5). AN1 lies in the domain targeted by MAbs 3C7 and 9/27 (reviewed in [40]). It has been shown that nAbs target epitopes on the C terminus of HVR1 and HVR1 is involved in SR-BI interaction [41, 42]. However, there is no evidence that antibodies against HVR1 are broadly cross-neutralising. Our amino acid substitution analysis also hints towards the strain specificity of VF-Fab. Nonetheless, why all four VF-Fabs were able to recognise the AN1 epitope is currently unclear (Table 6, S1 Fig 2A–2D). VF-Fab1a-1-3 recognised a second motif AN2_433-445_ which overlaps with motif AN3. A study by Deng *et al*. (2015) has shown that a major epitope lies on the neutralisation face of E2 glycoprotein, between amino acid 421–543 [15]. VF-Fab1b-5-1 and 3a-1-1 showed a higher affinity towards the β-turn mimics of epitope AN3_428-447._ AN3 is a part of the neutralisation face of E2 and intersects with the CBH-2 epitope as well [43]. AN3 also shares residues with HuMAb AR3C (Fig 5A, red solid boxes) [13]. Kong *et al*. (2013) have shown that N-terminal region residues 421 to 453 form a front layer a part of CD81 binding loop. The crystal structure of this epitope has shown that it is targeted by bNAbs [13]. This provides a probable explanation as to why VF-Fab1b-5-1 and VF-Fab3a-1-1 showed high affinity towards the β-turn mimics of epitope AN3 [13]. This higher affinity likely lead to the observed significant reduction of HCVpp infectivity when added in combination (Fig 6). VF-Fab1b-1-3, VF-Fab1b-5-1 and VF-Fab3a-1-1 have targeted a new epitope AN4 (residues 539–551). VF-Fab3a-1-1 also showed binding affinity towards epitope AN5_599-608_. AN4 amino acid residues 540 and 550 are involved in CD81 binding [13] however; AN4 and AN5’s role in HCV infection needs to be further characterised. Wong *et al*. (2014), mapped epitopes within E1E2 using antisera from humans immunised with recombinant E1E2 vaccine [11]. Wong and colleagues observed a strong competition between antisera from vaccinated humans (MF59C.1) with five well characterised MAb AP33 (CD81), AR3B (CD81), AR4A, AR5A, and IGH526 indicating the multi-epitope specific response by host immune system. Our epitope mapping results further provide important evidence that multiple motifs are targeted by host humoral immune system in the E2 glycoprotein. Therefore, our epitope mapping data answers the question that these patient derived VF-Fabs target multiple epitopes in HCVpp resulting in greater reduction in the infectivity.

The observed binding behaviour of these VF-Fabs suggests two possibilities a) VF- Fabs are of polyclonal nature, and/or b) they recognise discontinuous epitopes. The binding capacity of the VF-Fab pool represented here is inter-genotype and inter-subtype. Our immunovirology and bioinformatics analysis of amino acid variation of the putative epitopes predicted that the observed natural changes do not affect the functionality of the targets (Fig 4A, Table 4). SIFT calculates the probabilities for all the possible substitutions at each position and constructs a position-specific matrix considering the physiochemical properties of the amino acids based on the number of sequences provided. Positions with normalised probabilities <0.05 are predicted to be deleterious to the VF-Fab binding [44–46]. The observed variation in amino acid residues in the epitopes indicates that there must be preservation of the physicochemical properties and perhaps structural conformation to enable the VF-Fabs to target the previously “unseen” epitopes (probabilities range between 0.34–1.00). Specifically, this explains (in part) why VF-Fabs, which were never exposed to the unrelated E1E2 glycoprotein were still able to cross neutralise the HCVpp in our experiments (Figs 3 and 4).

A limitation of this study is that, we explored this phenomenon with only eleven patient derived VF-Fabs in genotype 1 and in an HCVpp system only. However, previous more limited studies and recent work by Wasilewski *et al*. (2016) collectively have shown that both HCVcc and HCVpp are equivalent with respect to the neutralisation phenotypes [5, 31, 47]. Nevertheless, despite this limitation, we present original and noteworthy findings in terms of the targeting of multiple epitopes, using unrelated patient derived VF-Fabs. Importantly, we have identified two new epitopes using VF-Fab obtained from immunologically active patients. Our immunovirological data lays a strong foundation for the investigation of humoral immune targeting of conserved HCV epitopes in antibody associated Hepatitis C virus.

## Supporting information

S1 Fig Total IgG from post proteinase K treated control sera are non-neutralising in nature. Total IgG from proteinase K treated human serum from male AB plasma from three different lots were used as control (SLBK465 V, SLBF2588V, 051M0919, Sigma). HCVpp incorporating E1E2 derived from genotype 1a (HCVpp1b-1-3, HCVppH77) and 1b (HCVpp1b-1-2, HCVpp1b-1-3) were pre-incubated with different concentrations (0.006 to 0.4 mg/ml) of control Total IgG prior to infection of Huh7 cells. A no envelope control was used to normalise the data. The neutralising activity is expressed as percentage of inhibition of the infectious titres. Each experiment was repeated three times. Error bars indicate standard deviation.
(TIF)

S2 Fig Heat map overview of peptides targeted by VF-Fab. Libraries of peptides beginning at the E2 N-terminus (residue 384–619 of the H77 reference strain AF011751) of the envelope protein were synthesized using chemically linked peptides on scaffolds (CLIPS) technology for conformational epitope mapping (Pepscan Presto; Lelystad, Netherlands).Individual peptides are listed on the right and VF-Fab are indicated at the base of the heatmap. Herceptin was used as an internal negative control and was screened with antibody 57.9 [48]. Native Cys were protected by acetamidomethyl in all the libraries (denoted by “2”). The magnitude of colour (dark magenta) with higher z score represents the binding affinity of VF-Fab to the peptide. All the VF-Fabs commonly bound peptides with core sequence _393_SRAAHRVTTFITR_405_ from all the sets. Additional binding was recorded for VF-Fab1a-1-3, VF-Fab1b-1-3 and VF-Fab1b-5-1 on linear peptides with core sequences _433_LNTGFLAALFYTH_445_ and _539_LLNNTRPPRGNWF_550_ respectively. VF-Fab1b-5-1 and VF-Fab31-1-1 similarly bound one β- turn mimic with core sequences _428_NCNDSLNTGFLAALFYTHRF_447_. Linear sequences _599_SGPWLTPRCM_608_, _539_LLNNTRPPRGNWF_550_ were additionally recognized by VF-Fab3a-1-1 (Table 3). Herceptin was used as an internal negative control. In order to make Heatmap legible, only every second peptide in the study has been included in the figure.
**A**. Linear peptides of 15 residues **B**. loop mimics of constrained peptides of 17 residues. **C**. structured peptides of 23 residues mimic the helical structure **D**. structured peptides of 22 residues mimic the β-turn.
(TIFF)
