# Supplementary figures and images for: Reverse epitope mapping of the E2 glycoprotein in antibody associated hepatitis C virus
Source: PLoS One. 2017 May 30;12(5):e0175349. doi: 10.1371/journal.pone.0175349 (PMC5448734; doi:10.1371/journal.pone.0175349)

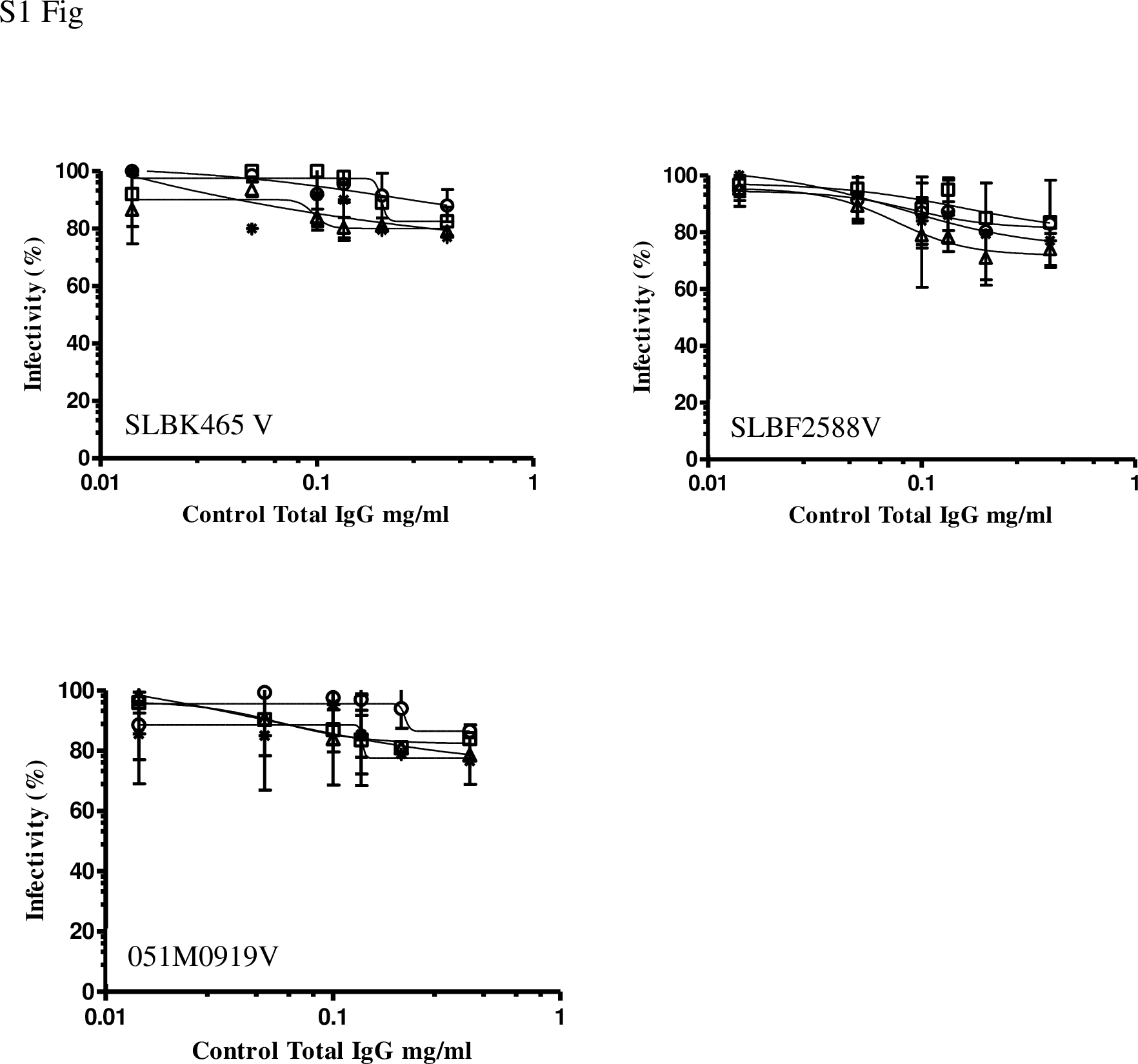

Supplement: S1 Fig — Total IgG from proteinase K treated human serum from male AB plasma from three different lots were used as control (SLBK465 V, SLBF2588V, 051M0919, Sigma). HCVpp incorporating E1E2 derived from genotype 1a (HCVpp1b-1-3, HCVppH77) and 1b (HCVpp1b-1-2, HCVpp1b-1-3) were pre-incubated with different concentrations (0.006 to 0.4 mg/ml) of control Total IgG prior to infection of Huh7 cells. A no envelope control was used to normalise the data. The neutralising activity is expressed as percentage of inhibition of the infectious titres. Each experiment was repeated three times. Error bars indicate standard deviation. (TIF) [file pone.0175349.s001.tif]

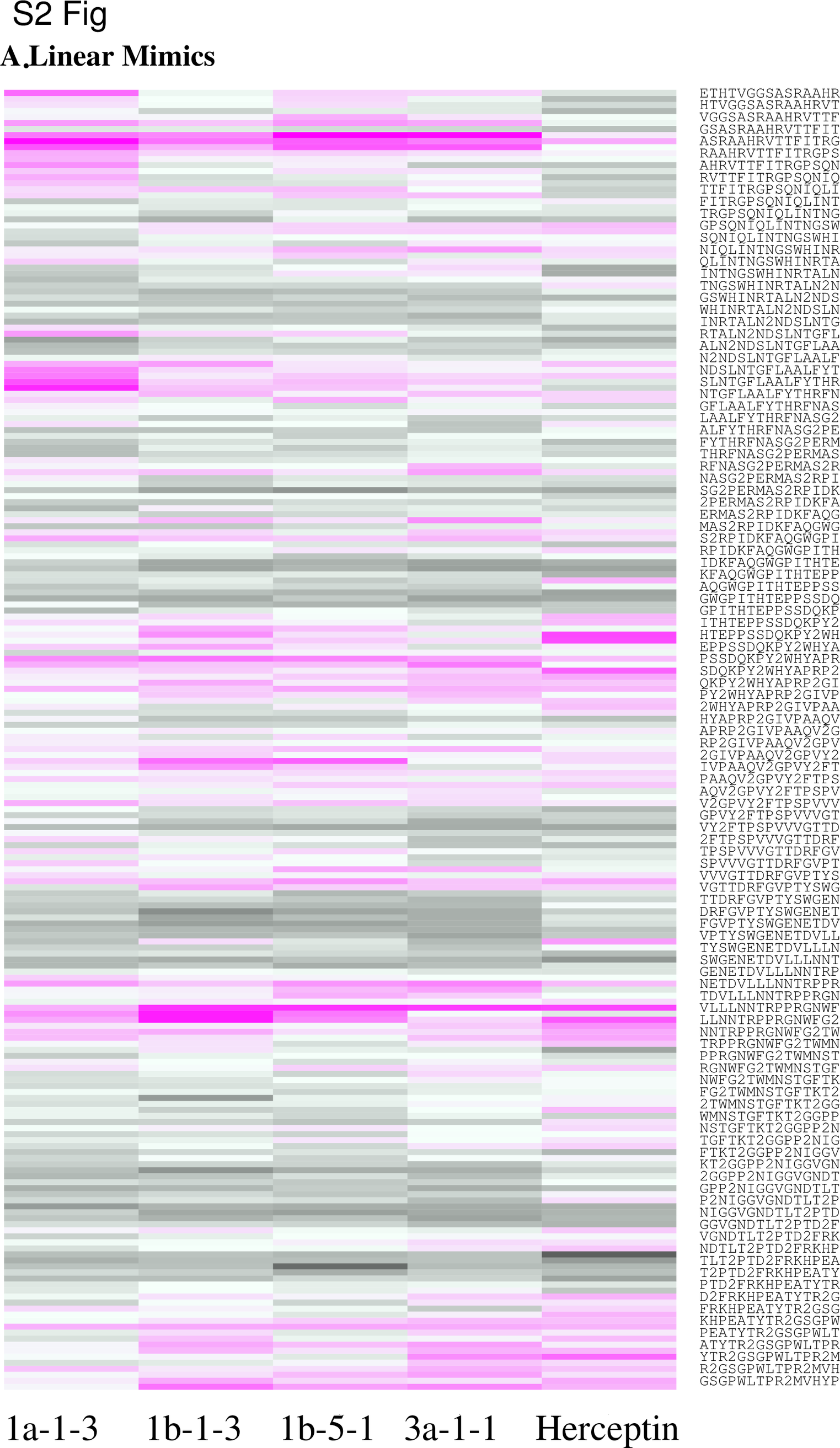

Supplement: S2 Fig — Libraries of peptides beginning at the E2 N-terminus (residue 384–619 of the H77 reference strain AF011751) of the envelope protein were synthesized using chemically linked peptides on scaffolds (CLIPS) technology for conformational epitope mapping (Pepscan Presto; Lelystad, Netherlands).Individual peptides are listed on the right and VF-Fab are indicated at the base of the heatmap. Herceptin was used as an internal negative control and was screened with antibody 57.9 [48]. Native Cys were protected by acetamidomethyl in all the libraries (denoted by “2”). The magnitude of colour (dark magenta) with higher z score represents the binding affinity of VF-Fab to the peptide. All the VF-Fabs commonly bound peptides with core sequence 393SRAAHRVTTFITR405 from all the sets. Additional binding was recorded for VF-Fab1a-1-3, VF-Fab1b-1-3 and VF-Fab1b-5-1 on linear peptides with core sequences 433LNTGFLAALFYTH445 and 539LLNNTRPPRGNWF550 respectively. VF-Fab1b-5-1 and VF-Fab31-1-1 similarly bound one β- turn mimic with core sequences 428NCNDSLNTGFLAALFYTHRF447. Linear sequences 599SGPWLTPRCM608, 539LLNNTRPPRGNWF550 were additionally recognized by VF-Fab3a-1-1 (Table 3). Herceptin was used as an internal negative control. In order to make Heatmap legible, only every second peptide in the study has been included in the figure. A. Linear peptides of 15 residues B. loop mimics of constrained peptides of 17 residues. C. structured peptides of 23 residues mimic the helical structure D. structured peptides of 22 residues mimic the β-turn. (TIFF) [file pone.0175349.s002.tiff]
